# Hand-Book of Diseases of the Ear

**Published:** 1904-06

**Authors:** 


**Hand-book of Diseases of the Ear.** By RICHARD LAKE. Pp. x., 232. London: Baillière, Tindall & Cox. 1903.

Students and practitioners will find in this handy volume a short but useful and thoroughly practical account of the various diseases which affect the ear and their treatment.

The removal of post-nasal adenoids is recommended "as soon as their presence is noted to any material extent" for aural prophylaxis. We can only accept this recommendation on the hypothesis that their presence will not be "noted" unless they cause obvious aural troubles or nasal obstruction. The views expressed with regard to the treatment of aural affections by intra-nasal operations are in accord with those held by the majority of English aural surgeons as expressed in recent discussions on the subject in the meetings of societies. Briefly stated, these views are that if aural inflation fails to relieve deafness, then intra-nasal operations will also fail. If, however, inflation ameliorates the condition, then intra-nasal operations will sometimes help to render the improvement more lasting.

The signs, symptoms, and treatment of chronic catarrhal otitis media and oto-sclerosis are well described. In the section on chronic suppurative otitis media we miss a paragraph giving succinct recommendations for ordinary treatment, though we admit that most of the ground is covered in subsequent sections which deal with the complications of chronic suppuration.

Ossiculectomy, with removal of the outer attic wall, for chronic suppuration in the middle ear, is said to be chiefly indicated in those patients who cannot give up the necessary time to undergo the radical mastoid operation, and the procedure is stated to yield extremely satisfactory results. We are of opinion that this is a great deal more than can be said for ossiculectomy without removal of the outer attic wall, and that if the results are as good as stated the indications for the operation are not adequately expressed.

We are interested in noting that the author, when operating for lateral sinus thrombosis, does not ligature the jugular vein unless he fails to get hemorrhage from the jugular end of the sinus by curetting in that direction through the opening in the sinus. We entirely agree with him in this matter. It is not necessary to tie the vein as a matter of routine in all cases.

We cannot congratulate the author on the artistic excellence of the three coloured plates which are provided to illustrate various pathological conditions of the membrana tympani, though they will prove of use to the beginner. A better sketch of the posterior nares or of the post-rhinoscopic image might have been given.

We know of no English book on aural diseases, of a comparable size, which we can more strongly recommend to the attention of students and practitioners.

